# Supplementary figures and images for: Generation of Population-Level Diversity in Anaplasma phagocytophilum msp2/p44 Gene Repertoires Through Recombination
Source: Pathogens. 2025 Feb 27;14(3):233. doi: 10.3390/pathogens14030233 (PMC11946796; doi:10.3390/pathogens14030233)

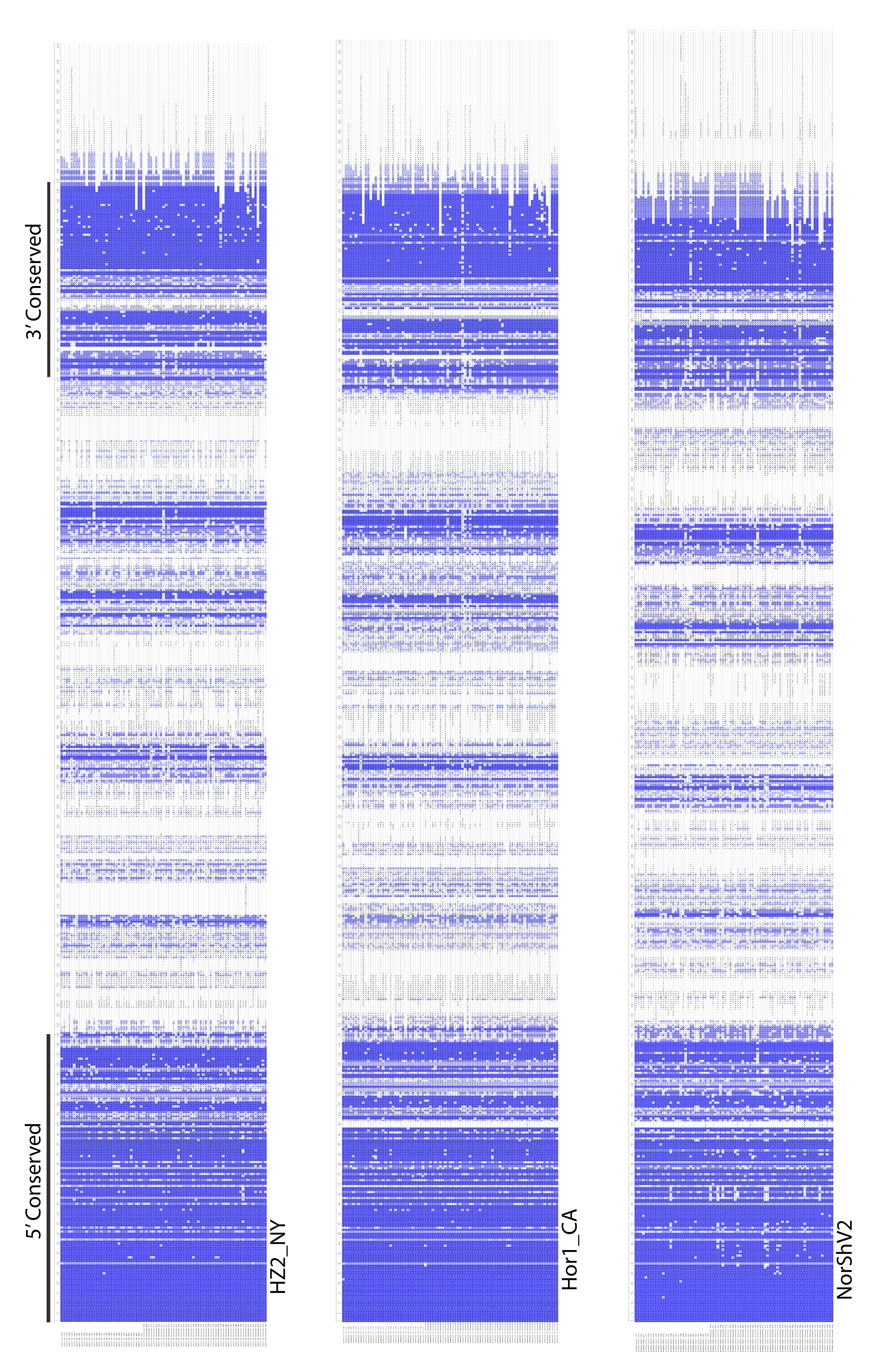

Supplement: Supplementary file 1 [file pathogens-14-00233-s001.zip › Supplementary Figure 1.png]

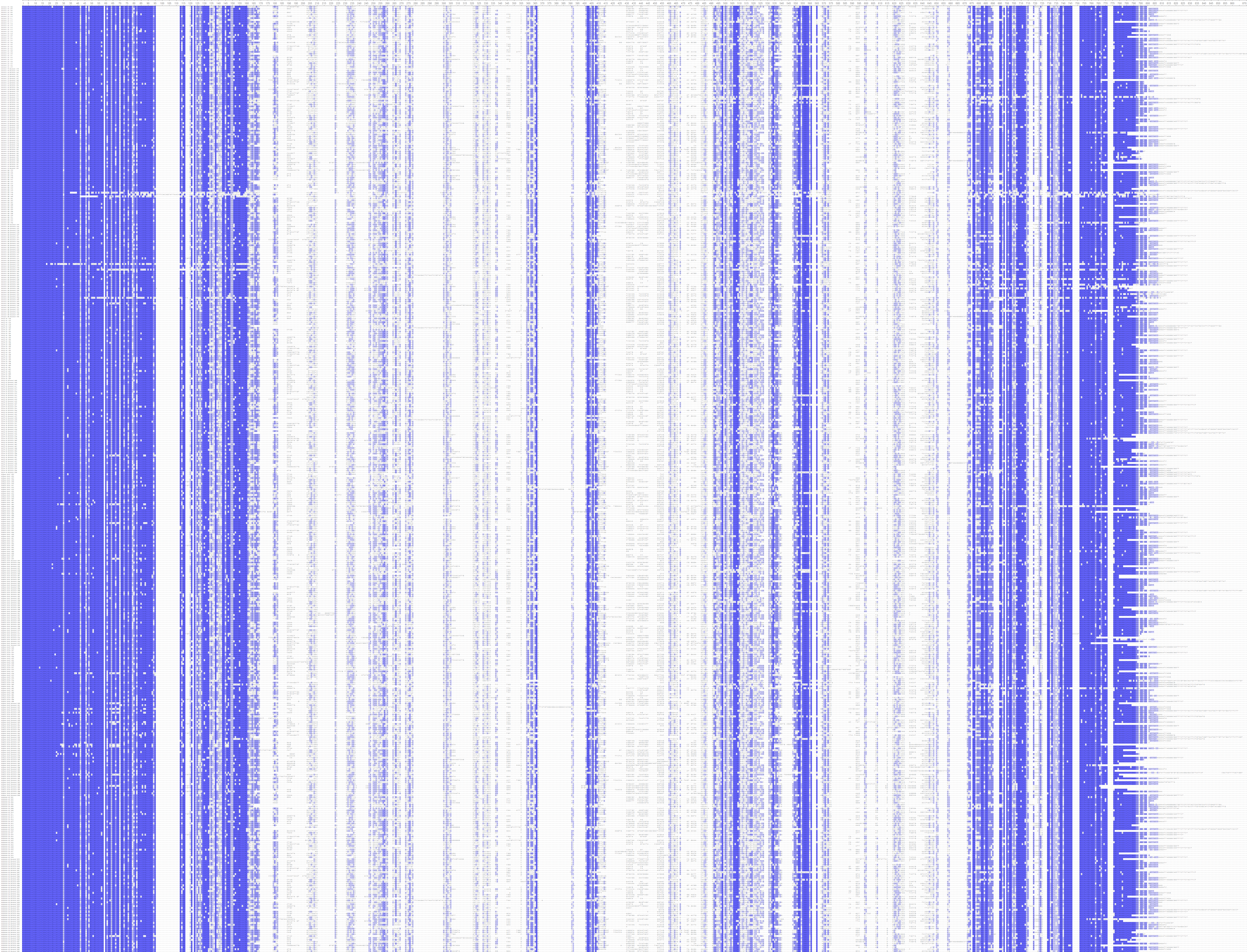

Supplement: Supplementary file 1 [file pathogens-14-00233-s001.zip › Supplementary Figure 2 Ap_all_alignment.png]
